# Clinical phenotypes of neuropathic pain and central sensitization in patients with knee osteoarthritis: demographic data, radiographic severity, and pain intensity

**DOI:** 10.1186/s12891-026-09747-4

**Published:** 2026-04-25

**Authors:** Mitsuhiko Kubo, Yuki Nosaka, Kosuke Kumagai, Yasutaka Amano, Eiji Isoya, Shinji Imai

**Affiliations:** 1 Department of Sports and Musculoskeletal Pain Medicine, Shiga University of Medical Science, Seta, Tsukinowa-cho, Otsu-City, Shiga Prefecture Japan; 2 Department of Orthopaedic Surgery, Jinseikai Kohnan Hospital, Koka-City, Japan; 3 Department of Orthopaedic Surgery, Shiga University of Medical Science, Otsu-City, Japan

**Keywords:** Knee osteoarthritis, Pain mechanism, Neuropathic pain, Central sensitization, Pain at rest

## Abstract

**Background:**

We aimed to simultaneously assess neuropathic pain (NP) and central sensitization (CS) in patients with knee osteoarthritis to determine their prevalence and identify patient characteristics associated with NP and CS based on demographic data, pain intensity, and radiographic severity commonly assessed in routine clinical practice.

**Methods:**

Patients with knee osteoarthritis (136) were evaluated. NP was assessed using the painDETECT questionnaire, with scores ≥ 13 categorized as NP( +). CS was assessed using the Central Sensitization Inventory (CSI), with scores ≥ 30 classified as CS( +). Pain intensity was measured at rest and during exercise using the visual analogue scale. Patients were grouped into low-pain and high-pain categories based on the average visual analogue scale score of pain at rest. Radiographic severity was assessed using the Kellgren-Lawrence grading system, classifying patients into low-grade (II–III) and high-grade (IV) categories. Demographic data, pain intensity, radiographic severity, painDETECT, and CSI scores were compared between the low- and high-pain intensity, low- and high-grade severity, NP( +) and NP( −), and CS( +) and CS( −) groups.

**Results:**

NP and CS were identified in 15.4% and 16.2% of patients, respectively, and either NP or CS mechanisms were observed in 25.6%. CSI scores were higher in the high-pain group, and pain at rest was significantly higher in NP( +) and CS( +) groups.

**Conclusions:**

Either NP or CS mechanisms were identified in 25.6% of patients with knee OA. Higher pain at rest was associated with NP and CS, particularly CS, in this cohort.

## Background

Pain is the primary symptom and leading cause of disability in patients with knee osteoarthritis (OA). Traditionally, the pain mechanism in knee OA has been regarded as purely nociceptive, arising from the activation of nociceptors due to structural damage or inflammation. However, the correlation between pain intensity and radiographic severity is only modest [1], suggesting that additional pain mechanisms contribute to the pain experience in knee OA. In addition to nociceptive pain, the International Association for the Study of Pain has defined neuropathic and nociplastic pains. Both neuropathic pain (NP) and nociplastic pain may be involved in some individuals with knee OA [2–9]. Moreover, patients with knee OA who exhibit these mechanisms have been reported to experience poor outcomes despite standard treatments such as nonsteroidal anti-inflammatory drugs [3], exercise therapy [4], and total knee arthroplasty (TKA) [5].

NP in patients with knee OA is commonly evaluated using questionnaires such as the painDETECT [10]. The assessment of nociplastic pain is more challenging, as it cannot not be measured directly. Central sensitization (CS), which can be evaluated using quantitative sensory testing or the Central Sensitization Inventory (CSI) [11], is widely recognized as the key mechanism underlying nociplastic pain and is often used as a surrogate measure. Despite these methods, few studies have simultaneously evaluated NP and CS in knee OA [12–14]. Furthermore, it remains unclear which patients are most affected by these pain mechanisms. Clarifying these characteristics may allow for the phenotypic classification of patients with knee OA based on pain mechanisms, thereby facilitating more individualized treatment strategies.

In this context, the present study aimed to characterize NP-like and CS-related symptom phenotypes in knee OA by simultaneously assessing NP with the painDETECT questionnaire and CS with the CSI. Specifically, we sought to (1) determine the prevalence of NP and CS and (2) examine clinical factors associated with these phenotypes, including demographics (age, sex, BMI), radiographic severity (Kellgren–Lawrence grade), and pain intensity measured separately at rest and during exercise using the visual analogue scale (VAS).

## Methods

### Patients

We evaluated 151 patients with knee OA grade II or higher on K–L grade (101 women and 35 men) who visited our clinic. Patients with incomplete survey responses (*n* = 2) and those taking oral medications considered to affect NP or CS (*n* = 13; duloxetine 7, pregabalin 5, and tramadol 1) were excluded. The study finally included 136 patients with knee OA (101 women and 35 men). The mean age of the participants was 72.8 years (range, 44–96 years), and the average BMI was 26.4 kg/m^2^ (range, 17.7–41.5 kg/m^2^). This study was approved by the institutional review board of Jinseikai Konan Hospital (reference number 2020–00), and the requirement for written informed consent was waived due to the retrospective nature of the study and the absence of identifiable participant information.

### Clinical and radiographic assessment

NP was evaluated using the painDETECT questionnaire, and CS, which is considered a key factor in nociplastic pain, was assessed using the CSI. The painDETECT is a self-administered questionnaire curated to identify NP. The score ranges from − 1 to 38, with the following proposed cutoffs: a score of ≤ 12 indicates that a neuropathic component is unlikely; ≥ 19, a neuropathic component is likely; and, 13–18, a possible neuropathic component [10]. Therefore, a patient with a painDETECT score of ≥ 13 was classified as having an NP component (NP +). Therefore, this definition includes both 'possible' (13–18) and 'likely' (≥ 19) neuropathic components, which may introduce some heterogeneity. Moreover, the CSI is a self-administered questionnaire used to assess CS. Based on the original version of the CSI by Mayer et al. [11], the Japanese version was developed and its validity and reliability have been verified [15]. The score ranges from 0 to 100, and patients with a CSI score ≥ 30 were defined as having CS (CS +) [16]. The VAS was used to evaluate pain intensity both at rest and during exercise. The scale consists of a 10-cm-long horizontal line, with one end representing no pain and the other representing intolerable pain. Patients were instructed to mark the line at a point that best represented their pain intensity. Based on their VAS scores of pain at rest, patients were divided into two groups: those with below-the-average and above-the-average VAS scores of pain at rest were classified as the low-pain and high-pain groups, respectively. This dichotomization was used for descriptive subgroup comparisons, and associations were additionally examined using continuous analyses and multivariable regression models. Additionally, standing knee anterior–posterior radiography was performed, and radiographic severity was classified according to the K-L grading system. Grades II–III were categorized as low-grade, and grade IV was categorized as high-grade radiographic severity.

### Comparisons between patient subgroups

Demographic factors, K–L grade, VAS scores (at rest/during exercise), painDETECT, and CSI scores were compared between the following subgroups: low-pain versus high-pain, low-grade versus high-grade radiographic severity, NP(−) versus NP(+), and CS(−) versus CS(+).

### Association analyses

Correlations between pain intensity (VAS at rest and during exercise) and painDETECT/CSI scores were assessed. In addition, multivariable logistic regression analyses were performed to examine the associations of pain intensity with CSI positivity (CSI ≥ 30) and painDETECT positivity (painDETECT ≥ 13), adjusting for age, sex, BMI, and K–L grade.

### Statistical analysis

A Mann–Whitney U test was used for subgroup comparisons. A P-value of < 0.05 was considered statistically significant. Receiver operating characteristic (ROC) curve analysis was performed for variables that showed significant between-group differences in the NP(+) versus NP(−) and CS(+) versus CS(−) comparisons; cutoffs were determined using Youden’s index. Spearman rank correlation coefficient (ρ) was used to assess correlations between pain VAS (at rest/during exercise) and painDETECT/CSI scores. Multivariable logistic regression analyses were performed with CSI positivity (CSI ≥ 30) and painDETECT positivity (painDETECT ≥ 13) as dependent variables, adjusting for age, sex, BMI, and K–L grade (modeled per 1-grade increase). Separate models were fitted for pain at rest and pain during exercise to avoid potential collinearity. Adjusted odds ratios (ORs) with 95% confidence intervals (CIs) are reported.

## Results

Patients’ demographic factors are shown in Table 1. Average VAS score of pain at rest was 1.9. Based on this, 89 and 47 cases were classified as low-pain and high-pain, respectively. According to the K–L grading system, there were 32 cases of grade II; 41, grade III; and, 63, grade IV. Based on this, 73 and 63 cases were classified as low-grade and high-grade, respectively.

**Table 1:** Patients’ demographic factors

	**Average (range)**
Sex (female/male)	101/35
Age	72.8 years (44–96)
Body mass index	26.4 kg/m^2^ (17.7–41.5)
Kellgren–Lawrence grade	II 32/III 41/IV 63
Pain at rest	1.9 (0–9.9)
Pain during exercise	6.1 (0–10)
PainDETECT	7.8 (− 1–22)
Central Sensitization Inventory	19 (2–55)

NP and CS were simultaneously assessed using the painDETECT questionnaire and the CSI to determine their prevalence in patients with knee OA. The average painDETECT score was 7.8 (range, − 1–22), with 21 cases (15.4% of patients) showing a NP component (NP +). The average CSI score was 19 (range, 2–55), with 22 cases (16.2% of patients) showing CS (CS +) (Table 1). Either NP or CS was involved in 25.6% of patients, leaving 74.4% with purely nociceptive pain.

We investigated patient characteristics associated with NP and CS based on demographic data, pain intensity, and radiographic severity commonly assessed in routine clinical practice. At first, we compared the painDETECT and the CSI scores between the low-pain and high-pain groups to evaluate the effect of pain intensity. No significant difference was found in painDETECT scores (low-pain: 7.3 ± 8.8, high-pain: 8.8 ± 4.4; *P* = 0.06). However, there was a significant difference in CSI scores (low-pain: 16.8 ± 9.3, high-pain: 28.7 ± 12.7; *P* = 0.001) (Table 2). Subsequently, we compared NP and CS between the low-grade and high-grade groups to examine the impact of radiographic severity. No significant differences were observed in the painDETECT (low-grade: 7.1 ± 4.8, high-grade: 8.6 ± 5.1; *P* = 0.08) and CSI scores (low-grade: 18.8 ± 9.8, high-grade: 18.9 ± 10.9; *P* = 0.76). Furthermore, no significant differences were observed in the VAS scores for pain at rest (low-grade: 1.8 ± 2.2, high-grade: 2.0 ± 2.3; *P* = 0.72). However, there was a significant difference in VAS scores for pain during exercise (low-grade: 5.7 ± 2.8, high-grade: 6.7 ± 2.5; *P* = 0.04) (Table 3).

**Table 2:** Comparison between low-pain and high-pain groups

	Low-pain group (n = 89)	High-pain group (n = 47)	***P*-Value**
Sex(female/male)	63/26	38/9	0.2
**Age**	**74.1 ± 9.5**	**70.2 ± 9.9**	0.03*
Body mass index	26.2 ± 4.2	26.7 ± 4.9	0.41
Kellgren–Lawrence grade	48/41	24/23	0.75
PainDETECT	7.3 ± 8.8	8.8 ± 4.4	0.06
**Central Sensitization Inventory**	**16.8 ± 9.3**	**28.7 ± 12.7**	0.001*

Values are mean ± SD unless otherwise indicated

Low-pain: VAS score of pain at rest < average score, high-pain: VAS score pain at rest ≧ average score

**Table 3:** Comparison between low-grade and high-grade groups

	Low-grade (*n* = 73)	High-grade (*n* = 63)	***P*-value**
Sex (female/male)	50/23	51/12	0.1
**Age**	**69.0 ± 10.1**	**77.2 ± 7.3**	< 0.001*
Body mass index	26.0 ± 4.7	26.8 ± 4.2	0.25
Pain at rest	1.8 ± 2.2	2.0 ± 2.3	0.72
**Pain during exercise**	**5.7 ± 2.8**	**6.7 ± 2.5**	0.04*
painDETECT	7.1 ± 4.8	8.6 ± 5.1	0.08
Central Sensitization Inventory	18.8 ± 9.8	18.9 ± 10.9	0.76

Values are mean ± SD unless otherwise indicated

Low-grade: Kellgren–Lawrence grade II and III, high-grade: Kellgren–Lawrence grade IV

^*^Significant difference: Mann–Whitney U test

Finally, demographic factors, pain intensity, and radiographic severity were compared between patients in whom NP and CS were involved and those in whom they were not. When comparing the NP(−) and NP(+) groups, a significant difference was observed in VAS scores for pain at rest (NP(−): 1.7 ± 2.1, NP(+): 3.0 ± 2.8; *P* = 0.02). When comparing the CS(−) and CS(+) groups, a significant difference was observed in VAS scores for pain at rest (CS(−): 1.7 ± 2.2, CS(+): 3.2 ± 2.4; *P* = 0.003) (Tables 4 and 5). In continuous analyses, the pain VAS score at rest correlated with both painDETECT (ρ = 0.31, *P* < 0.001) and CSI (ρ = 0.26, *P* = 0.003) scores, and the pain VAS score during exercise correlated with painDETECT (ρ = 0.28, *P* = 0.001) but not with CSI (ρ = 0.16, *P* = 0.060) (Table 6) scores. In multivariable logistic regression adjusting for age, sex, BMI, and K–L grade, the pain VAS score at rest remained associated with CSI positivity (adjusted OR 1.31 per 1-point increase, 95% CI 1.08–1.58; *P* = 0.006) and painDETECT positivity (adjusted OR 1.31, 95% CI 1.07–1.60; *P* = 0.008), whereas that during exercise was not significantly associated with CSI or painDETECT positivity (Tables 7 and 8). ROC analyses showed modest discrimination (AUC 0.65 for NP and 0.70 for CS); therefore, the derived cutoffs (VAS ≥ 1 for NP and ≥ 2.1 for CS) should be interpreted as cohort-specific indicative values rather than clinically actionable thresholds (Table 9).

**Table 4:** Comparison between NP(−) and NP(+) groups

	NP (−) (*n* = 115)	NP (+) (*n* = 21)	***P*-value**
Sex (female/male)	84/31	17/4	ns
Age	72.2 ± 9.8	75.9 ± 9.2	0.13
Body mass index	26.3 ± 4.4	26.9 ± 4.6	0.41
Kellgren–Lawrence grade (II–III/IV)	64/51	9/12	ns
**Pain at rest**	**1.7 ± 2.1**	**3.0 ± 2.8**	0.02*
Pain during exercise	6.0 ± 2.7	6.9 ± 3.0	0.10

Values are mean ± SD unless otherwise indicated

^*^Significant difference: Mann–Whitney U test

**Table 5:** Comparison between CS(−) and CS(+) groups

	CS (−) (*n* = 114)	CS (+) (*n* = 22)	***P*-value**
Sex (female/male)	83/31	18/4	Ns
Age	72.6 ± 10.0	73.6 ± 8.7	0.62
Body mass index	26.3 ± 4.2	26.8 ± 5.5	0.66
Kellgren–Lawrence grade (II–III/IV)	62/52	11/11	Ns
**Pain at rest**	**1.7 ± 2.2**	**3.2 ± 2.4**	0.003*
Pain during exercise	6.0 ± 2.8	7.0 ± 2.1	0.12

Values are mean ± SD unless otherwise indicated

^*^Significant difference: Mann–Whitney U test

**Table 6:** Correlation between pain VAS and painDETECT and CSI

	painDETECT (ρ)	*P*-value	CSI (ρ)	*P*-value
Pain at rest	0.31	< 0.001	0.26	0.003
Pain during exercise	0.28	0.001	0.16	0.060

Values are Spearman rank correlation coefficients (ρ)

*Abbreviations*: *VAS* visual analogue scale, *CSI* Central Sensitization Inventory

^*^P < 0.05

**Table 7:** Multivariable logistic regression for CSI positivity (CSI ≥ 30; *N* = 136; CSI + *n* = 22)

Predictor	Model 1: Pain at rest Adjusted OR (95% CI)	*P*-value	Model 2: Pain during exercise Adjusted OR (95% CI)	*P*-value
Sex (male vs female)	0.70 (0.21–2.37)	0.563	0.56 (0.17–1.85)	0.344
K–L grade (per 1-grade increase)	0.85 (0.43–1.67)	0.628	0.79 (0.39–1.58)	0.498
Age (per 1 year)	1.04 (0.98–1.10)	0.227	1.03 (0.97–1.09)	0.363
BMI (per 1 kg/m^2^)	1.04 (0.93–1.15)	0.521	1.03 (0.93–1.15)	0.540
Pain VAS (per 1 point)	1.31 (1.08–1.58)	0.006	1.19 (0.98–1.44)	0.080

Outcome: CSI positivity defined as CSI ≥ 30 (CSI +). All covariates were entered simultaneously. K–L grade was modeled as a continuous variable (per 1-grade increase). ORs are expressed per 1-unit increase unless otherwise specified. Separate models were fitted for pain at rest and pain during exercise to avoid potential collinearity

*Abbreviations*: *OR* odds ratio, *CI* confidence interval, *VAS* visual analogue scale, *BMI* body mass index, *K–L* Kellgren–Lawrence

**Table 8:** Multivariable logistic regression for painDETECT positivity (painDETECT ≥ 13; N = 136; painDETECT + *n* = 21)

Predictor	Model 3: Pain at rest Adjusted OR (95% CI)	P-value	Model 4: Pain during exercise Adjusted OR (95% CI)	P-value
Sex (male vs female)	0.74 (0.21–2.57)	0.631	0.59 (0.18–2.01)	0.403
K–L grade (per 1-grade increase)	1.02 (0.50–2.09)	0.965	0.97 (0.47–2.01)	0.928
Age (per 1 year)	1.07 (1.00–1.14)	0.050	1.05 (0.99–1.12)	0.095
BMI (per 1 kg/m^2^)	1.05 (0.94–1.18)	0.371	1.05 (0.94–1.18)	0.380
Pain VAS score (per 1 point)	1.31 (1.07–1.60)	0.008	1.15 (0.95–1.40)	0.152

Outcome: painDETECT positivity defined as painDETECT ≥ 13 (painDETECT +). All covariates were entered simultaneously. K–L grade was modeled as a continuous variable (per 1-grade increase). ORs are expressed per 1-unit increase unless otherwise specified. Separate models were fitted for pain at rest and pain during exercise to avoid potential collinearity

*Abbreviations*: *OR* odds ratio, *CI* confidence interval, *VAS* visual analogue scale, *BMI* body mass index, *K–L* Kellgren–Lawrence

**Table 9:** Receiver operating characteristic curve analysis

	Cut-off point	Odds ratio (95% CI)	P-value	AUC (95% CI)	**AUC**
Neuropathic pain	Pain at rest: 1	4.8 (1.16–19.79)	0.03	0.65 (0.52–0.78)	0.65
Central sensitization	Pain at rest: 2.1	4.9 (1.72–14.00)	0.003	0.70 (0.58–0.82)	0.70

*Abbreviations*: *AUC* Area Under the Curve

## Discussion

Pain in knee OA has traditionally been considered nociceptive, resulting from structural damage or inflammation. However, accumulating evidence indicates that NP and CS also contribute to knee OA pain. Patients affected by NP or CS have been reported to exhibit poorer outcomes despite standard treatments, underscoring the clinical relevance of characterizing these mechanisms. Thus, clarifying not only the prevalence of NP and CS in knee OA but also the clinical characteristics of patients in whom these mechanisms may be involved is important. Nevertheless, few studies have simultaneously evaluated NP and CS in knee OA, and the prevalence and clinical characteristics of affected patients remain insufficiently understood.

**Fig. 1:**
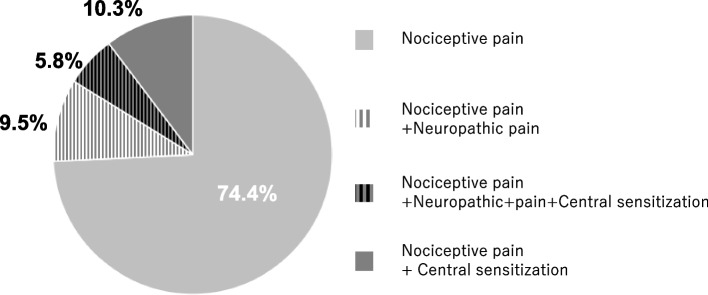
Distribution of pain phenotypes among patients with knee osteoarthritis. The proportions of nociceptive pain alone and various combinations involving neuropathic pain and central sensitization are shown. NP was defined as a painDETECT score ≥ 13, and CS was defined as a CSI score ≥ 30

The present study simultaneously assessed NP and CS and identified their prevalence and associated characteristics based on demographic data, pain intensity, and radiographic severity commonly assessed in routine clinical practice. The first objective of this study was to simultaneously evaluate the prevalence of NP and CS in patients with knee OA. NP was observed in 15.4% of patients, and CS was observed in 16.2%. Either NP or CS was identified in 25.6% of patients, indicating that only 74.4% of the patients had purely nociceptive pain (Fig. 1).

### Prevalence of NP and CS

The first report on the prevalence of NP in knee OA using the painDETECT questionnaire, a commonly used evaluation method that was also employed in the present study, was published by Hochman et al. [6]. They found that 19% of patients with painDETECT scores ≥ 19 had NP. A meta-analysis reported that the prevalence of NP in knee OA ranges from 5.4% to 51.9% [7]. CS, which is believed to play a central role in nociplastic pain, is typically measured using the CSI. Kim et al. were the first to investigate CS in patients with knee OA using the CSI and reported that 48.3% of patients had a CSI score greater than 40 [8]. A recent meta-analysis found a prevalence of CS in knee OA of 33% (range 24.8–48.3%) [9].

### Simultaneous evaluation of the prevalence of NP and CS

Only three studies have simultaneously evaluated the prevalence of NP and CS in knee OA, as far as we have been able to ascertain [12–14]. Goldoni et al. reported that, among outpatients with knee OA, 40.8% had painDETECT scores > 19, and 48% had CSI scores > 40, with 62.4% of patients exhibiting at least one of these components [12]. Ogawa et al. reported that, among patients with knee OA scheduled for knee arthroplasty or osteotomy, 17.3% had painDETECT scores > 12, 8.1% had CSI scores > 40, and 22.4% of patients had at least one of these components [13]. Kim et al. reported that, among patients with knee OA scheduled for TKA, 28.5% had painDETECT scores > 19, and 38.9% had CSI scores > 40, with 50% of patients exhibiting at least one of these components [14]. In the present study, we evaluated NP based on painDETECT scores greater than 12 and CS based on CSI scores greater than 30 in outpatients with knee OA. NP was present in 15.3% of patients, CS in 16.1%, and either component among NP and CS in 25.6% (Fig. 1).

Phenotypic classification of patients based on pain mechanisms may help describe heterogeneity in knee OA pain and support hypothesis generation for future studies. Therefore, understanding the clinical characteristics of patients who exhibit these pain mechanisms is important for phenotypic characterization, rather than for making direct treatment recommendations based on this cross-sectional study. These findings may inform the design of future longitudinal and interventional studies evaluating temporal relationships and treatment response in patients with NP-like and/or CS-related symptoms.

The second objective of this study was to explore patient characteristics associated with NP and CS based on demographic data, pain intensity, and radiographic severity commonly assessed in routine clinical practice. Pain at rest was higher in patients with NP or CS, and the pain VAS score at rest showed modest correlations with painDETECT and CSI scores. After adjustment for age, sex, BMI, and K–L grade, pain at rest remained associated with CSI positivity and painDETECT positivity, whereas pain during exercise was not significantly associated with either outcome (Tables 7 and 8). ROC analyses suggested cohort-specific indicative cutoffs for pain at rest; however, discriminative performance was only fair (AUC 0.65–0.70), and these thresholds should not be interpreted as clinically actionable diagnostics. Collectively, higher pain at rest may raise suspicion of NP/CS features, particularly CS, but cannot reliably discriminate these mechanisms in isolation.

### Relationship of patients’ demographic factors in NP and CS

Several studies have examined the relationship between demographic data, such as age, sex, and BMI, and NP or CS [8, 17–22]. Many studies have reported that these demographic factors do not significantly affect the presence of these pain mechanisms, and our findings are consistent with this conclusion.

### Relationship of radiographic severity in NP and CS

Several studies have explored the relationship between NP and radiographic severity. Polat et al. [21] evaluated NP using painDETECT in patients with knee OA and classified them into three groups: likely NP (score > 19), possible NP (score between 13 and 18), and unlikely NP (score < 12). As no difference was observed in K–L grade ratios among these groups, they concluded that NP is not associated with OA stage. Recently, Ogawa et al. used painDETECT to classify patients as having possible or unlikely NP and compared radiological findings, including X-rays and MRI, and found no significant differences [13]. Few studies have examined the relationship between radiographic severity and CS as assessed using the CSI. Kim et al. assessed CS using the CSI in 98 patients scheduled for TKA and compared the K–L grade between patients with CSI scores greater than 40 and those with scores below 40, reporting no significant differences [8]. Sasaki et al. evaluated CS in 942 community-dwelling volunteers using the CSI-9 and found no significant differences across K-L grades [23]. A systematic review published in 2021 concluded that these pain mechanisms, namely NP and CS, are not associated with the structural severity of knee OA [24]. NP and CS can occur independently of radiographic severity.

In this study, there was no significant difference in painDETECT and CSI scores between patients with low-grade and high-grade radiographic severity. This result indicated that radiographic severity does not affect NP or CS and was consistent with previous reports. In our cohort, age differed between radiographic severity subgroups, and age may influence both radiographic severity and pain phenotypes. Therefore, the lack of association between radiographic severity and NP/CS measures should be interpreted with caution, as residual confounding by age and limited statistical power may partially mask underlying relationships.

### Relationship of pain intensity in NP and CS

The first report on the relationship between NP and pain intensity in knee OA was published by Hochman et al. [6]. They divided patients with OA into two groups based on painDETECT scores > 19 points and found that the Western Ontario and McMaster Universities Osteoarthritis Index (WOMAC) pain scores were significantly higher in patients with NP symptoms. Subsequently, several studies have reported that NP is related to VAS pain and WOMAC pain scores [13] and is correlated with pain at rest, as seen in our study [21, 22]. Kim et al. [8] investigated the relationship between CS and pain intensity in patients scheduled to undergo TKA. They evaluated CS using the CSI and found that patients with CSI scores > 40 had significantly higher knee pain intensity, as assessed by the Numeric Rating Scale (NRS). Moreover, Sasaki et al. [23] reported that nocturnal pain was more pronounced in patients with knee OA with CS.

In this study, CSI scores were significantly higher, and painDETECT scores tended to be higher in high-pain patients compared with low-pain patients. When comparing patients with and without NP and those with and without CS, pain at rest was significantly higher in patients with NP as well as in those with CS. Pain intensity correlates with NP and CS, and it is believed that NP and CS contribute to more severe pain, especially pain at rest.

### Difference in the relationship between pain mechanism and type of pain

Our results showed no significant difference in pain during exercise but a significant difference in pain at rest between the two groups of NP (+) and (−) and CS (+) and (−). The pain mechanism may differ depending on the type of pain, and pain at rest is an important indicator of NP and CS. Pain is a subjective experience and can be challenging to evaluate. Pain intensity can be assessed using scales such as the VAS or NRS or through multi-question assessments such as WOMAC. WOMAC has the advantage of comprehensively assessing pain in various situations, such as walking, stair climbing, nocturnal pain, rest, and weight-bearing, whereas VAS and NRS offer the advantage of evaluating pain at rest, pain during exercise, and other target pain separately, as done in this study. There are reports indicating that no significant correlation was found between pain at rest and pain during movement [25], with pain at rest often related to psychological factors and pain during exercise linked to mechanical factors [26]. Therefore, in the present study, we evaluated pain using two different VAS measures: pain at rest and pain during exercise. To the best of our knowledge, no study has clarified the relationship between the type of pain (pain at rest versus during exercise) and pain mechanisms (NP and CS) in knee OA, as in this study. Regarding the differences in the relationship between the type of pain and NP, Power et al. [22] reported that pain at rest affects NP more than pain during activity in women and that pain at rest, but not pain during activity, affects NP in men. Regarding the relationship between the type of pain and CS, pain at night/on sitting, that is, pain at rest in this study, among the five components of the WOMAC pain score, was significantly correlated with the pain threshold, that is, CS [26].

In this study, pain during exercise was significantly different between patients with high-grade or low-grade severity but not different between NP(+) and (−) and CS(+) and (−) groups. There are reports similar to ours in which movement-evoked pain (i.e., pain during exercise in this study) does not correlate with the CSI-9 [4]. In other words, pain during exercise is correlated with radiographic severity and not related to CS. Knee OA pain is thought to be difficult to interpret because it does not correlate well with radiographic severity. This discrepancy is thought to result from the influence of NP and CS mechanisms. Pain during exercise originates from the nociceptive pain mechanism, and pain at rest originates from the NP and CS mechanisms. By evaluating each type of pain separately according to its underlying mechanism, we can better understand the complex nature of knee OA pain.

### Limitations

The first limitation is the relatively small sample size and the single-center design, which may limit generalizability. Second, several variables were dichotomized for descriptive subgroup comparisons (e.g., low vs high pain based on the cohort mean), which may lead to information loss and cohort-dependent thresholds; therefore, we additionally examined associations using continuous correlations and multivariable logistic regression models. Third, the painDETECT score threshold of ≥ 13 includes both “possible” and “likely” neuropathic components; thus, the NP(+) group may be heterogeneous. Fourth, because patients receiving analgesics or other pain-modulating medications were excluded, the prevalence of painDETECT positivity and/or CSI positivity may have been underestimated. Accordingly, the reported prevalence should be interpreted as cohort-specific. Fifth, ROC-derived cutoffs were obtained from the same dataset and showed only fair discrimination; therefore, these values should be regarded as indicative within this cohort and require external validation. Finally, the cross-sectional design precludes inference on directionality, and the observed associations between pain at rest and NP-like symptoms/CS-related symptoms should be interpreted as non-causal and potentially bidirectional. Despite these limitations, our findings suggest that a substantial proportion of patients with knee OA exhibit NP-like and/or CS-related symptoms, and higher pain at rest may be a clinically relevant signal warranting further assessment.

## Conclusions

In patients with knee OA, 74.4% experienced pain consistent with a predominantly nociceptive mechanism. However, 25.6% of patients exhibited either NP or CS components. In this cross-sectional cohort, NP and CS were not associated with radiographic severity, whereas higher pain at rest was associated with NP and CS. These findings support phenotypic characterization and hypothesis generation; prospective studies are needed to clarify directionality and determine whether mechanism-based stratification improves clinical outcomes.

## Data Availability

The datasets are available from the corresponding author on reasonable request.

## Abbreviations

AUC: Area under the curve
CS: Central sensitization
CSI: Central Sensitization Inventory
K-L: Kellgren–Lawrence
NP: Neuropathic pain
NRS: Numeric Rating Scale
OA: Osteoarthritis
TKA: Total knee arthroplasty
VAS: Visual analogue scale
WOMAC: Western Ontario and McMaster Universities Osteoarthritis Index
